# *CA* turns 75: Looking at the future but never forgetting the roots

**DOI:** 10.3322/caac.70040

**Published:** 2025-11-03

**Authors:** Don S. Dizon, Sumanta Kumar Pal, Banu E. Symington, Razelle Kurzrock, Arif H. Kamal, Christina M. Annunziata, Shanthi Sivendran, Ahmedin Jemal, William L. Dahut

**Affiliations:** ^1^ Division of Hematology and Oncology Department of Medicine Tufts University School of Medicine Boston Massachusetts USA; ^2^ Department of Medical Oncology City of Hope Comprehensive Cancer Center Duarte California USA; ^3^ Sweetwater Regional Cancer Center Memorial Hospital of Sweetwater County Rock Springs Wyoming USA; ^4^ Division of Hematology and Medical Oncology Medical College of Wisconsin Cancer Center Milwaukee Wisconsin USA; ^5^ American Cancer Society Durham North Carolina USA; ^6^ Extramural Discovery Science American Cancer Society Glen Allen Virginia USA; ^7^ Cancer Treatment Support American Cancer Society Fort Washington Pennsylvania USA; ^8^ Surveillance and Health Equity Science American Cancer Society Atlanta Georgia USA; ^9^ American Cancer Society Chevy Chase Maryland USA

*CA: A Cancer Journal for Clinicians* (*CA*) began in 1950 as *CA: A Bulletin of Cancer Progress* (Figure 1) and, in 2025, celebrates its 75th year in continuous publication. Today, it is the flagship journal of the American Cancer Society (ACS) and is associated with the highest impact factor of any medical or scientific journal, inside and outside of oncology—a testament to its value, not only to professionals in this space but to the wider public at large. The core reason for its domestic and global reach is *cancer statistics*, a continuous and evolving effort to describe the incidence and mortality of cancers and how changes in exposures, diagnosis, and treatment affect them. We would be remiss if not acknowledging the 14 years of significant contributions made to the intellectual rigor and refined analyses of these reports, assembled under the guidance of Dr Ahmedin Jemal, Senior Vice President of the Surveillance and Health Equity Science Department.

**FIGURE 1:**
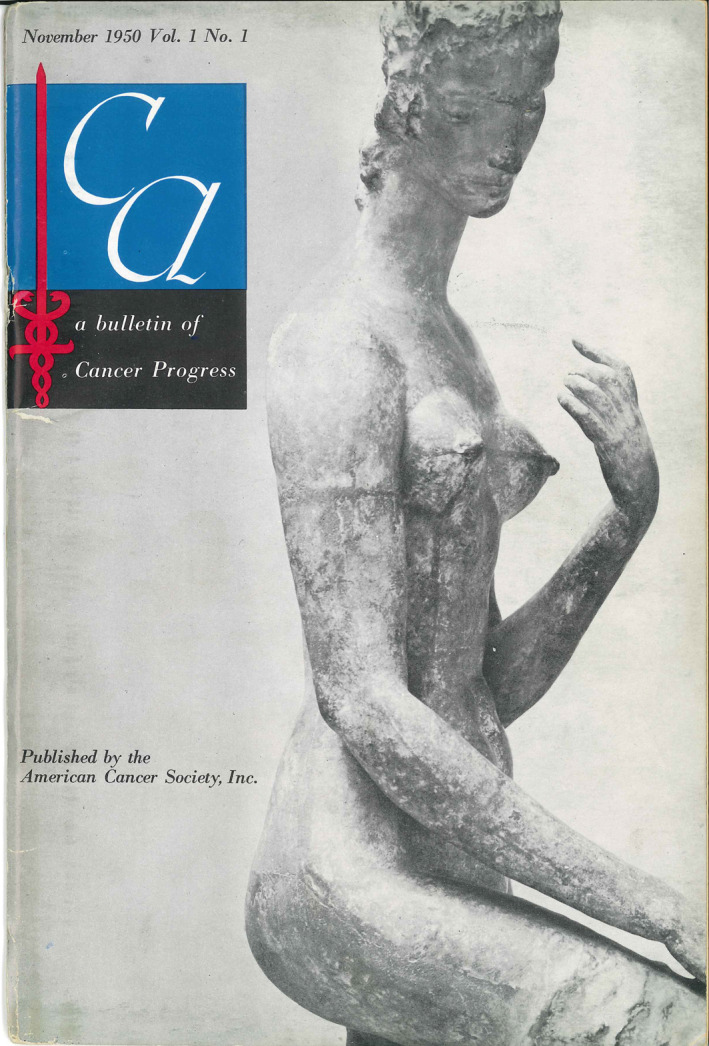
Cover of *CA: A Bulletin of Cancer Progress* volume 1, issue 1, November 1950.

Beyond cancer statistics, *CA* fulfills a major role: the dissemination of information about cancer across its continuum. It has provided our peers and the public contemporary and updated, expert, open‐access reviews, at no cost, actively demonstrating a core value of the ACS on the widespread dissemination of cancer practice, research, and education. Reflecting the collaboration of the editorial team and authors, these reviews serve as a resource for everyone and do not require specialization in oncology to comprehend them.

As *CA* celebrated its 60th year, the editorial team led by Dr Ted Gansler reflected on seminal publications during its first decade, from the Papanicolaou smear for the early detection of cervical cancer to prognostic disclosure in clinician–patient communication.^1^ It seems fitting then that, as members of the editorial board, we offer our own perspective as we highlight significant publications since that first decade. Finally, we collectively reflect on our mission as we move into the future.

## A RISING INCIDENCE OF COLORECTAL CANCER IN YOUNG ADULTS

Colorectal cancer (CRC) is the fourth most common diagnosed cancer and the second leading cause of cancer death in the United States, effecting over 150,000 people and accounting for over 50,000 deaths.^2^ In 2017, Siegel et al. reported increased CRC incidence rates in both men and women younger than 55 years, whereas rates continued to decline in aged 55 years or older.^3^ Consequently, the proportion of CRC being diagnosed in people younger than 55 years rose from 11% of all cases in 1995 to 20% in 2019.^2^ The increase in young‐onset CRC is a global phenomenon, with rates rising in parts of Europe, South America, Oceania, and Asia as well as in Canada.^4^, ^5^ Work is underway to understand the biologic and systemic factors that account for early onset CRC and ways to improve early detection and treatment.

## THE FACTOR YEARS SINCE QUITTING DOES NOT EXCLUDE ONE FROM SCREENING FOR LUNG CANCER

A testament to the work of the ACS is provided by the efforts in its Guideline Development Group, and none is more significant than those made to update its lung cancer screening guidelines to reflect contemporary analyses. First reported in 1980 as part of its cancer‐related health checkup,^6^ the first study of lung cancer screening with low‐dose computed tomography (LDCT) was published in 2013, reflecting a collaboration between the ACS, the American College of Chest Physicians, the American Society of Clinical Oncology, and the National Comprehensive Cancer Network.^7^ In 2023, the guidelines reflected one important consideration: *years since quitting* should be removed as a factor when offering LDCT screening. This guideline evolution stems from the understanding that, although the risk of subsequent lung cancer falls once someone quits smoking, it remains elevated after 20 and 30 years since quitting compared with the risk for someone who never smoked.^8^ This expands the population eligible for LDCT and ultimately may save many from death from lung cancer.

## USHERING IN THE ERA OF PERSONALIZED ONCOLOGY

In 2011, Maitland and Schilsky reflected on the emerging importance of molecular mechanisms in the clinical practice of oncology, which served as the basis to call for *equally innovative advances in the design and conduct of clinical trials*, written at a time that saw the development of trastuzumab in human epidermal growth factor receptor 2–positive breast cancer and the availability of oral tyrosine kinase inhibitors for Philadelphia chromosome–positive chronic myelogenous leukemia.^9^ Fourteen years later, we have ushered in the era of precision oncology, highlighted by the incorporation of biologic factors into cancer staging, first discussed in 2017^10^; solid tumor evaluation and management^11^, ^12^; and the approval of treatments based on biology rather than on the organ of origin (i.e., tissue‐agnostic therapies).^13^

## PATIENT NAVIGATION ACROSS THE CANCER TRAJECTORY: FROM RESEARCH TO PRACTICE

The concept of patient navigation is one that dates back to the 1990s, but early experiences lacked data on effectiveness across the cancer trajectory. In 2011, Paskett et al. reviewed the state of the science indicating that navigation favorably affected screening rates, particularly in breast cancer and CRC, whereas no clear benefit in treatment outcomes (e.g., adherence, health care utilization) was reported, pointing toward the need for more rigorous research.^14^ Since that time, much work has been done, as summarized in an umbrella review by Chan et al., who reported the evidence‐based benefits of navigation from screening to diagnosis, treatment, and in survivorship.^15^ The companion editorial was written by Paskett and colleagues as well, indicating that we had come full circle—sufficient data had been generated to *support* navigation, indicating that research should now move toward the *implementation* of navigation in usual care,^16^ a remarkable evolution of a topic whose development was documented in the pages of this journal.

## THE REAL‐WORLD EXPERIENCE OF CANCER TREATMENT: VIRTUAL TUMOR BOARDS

In 2020, Dr. Keith Delman initiated the virtual tumor boards in *CA*. Meant to reflect the multidisciplinary approach to the management of cancer, it has evolved into a forum to demonstrate the impact of multi‐omics testing in clinical practice while reflecting the lived experience of patients themselves.^17^ Virtual tumor boards cover the intricacies in treatment decision making and the role of novel technologies in daily management. They go beyond the sphere of investigational approaches and allow for readers across the world to witness how expert groups from various institutions work together to achieve quality care for the person with cancer.

## LOOKING INTO THE FUTURE

That the ACS made the decision to hire an Editor‐in‐Chief from outside its organization reflects on its desire to broaden the reach, scope, and access to the journal for authors and readers alike. Alongside an editorial team that includes ACS leadership, we are privileged to usher in this new era in *CA* through the opportunities to publish phase 3 trials that will inform standards of care and consensus guidelines offering expert viewpoints on the management of controversial areas across oncology. We aim to provide a venue for reviews from key opinion leaders across the globe to ensure that the relevance and access to its pages reaches everyone: oncologist and nononcologist alike. Much of what we cover summarizes the evolving trends in oncology, particularly as it relates to the biologic basis of neoplasia and the way we treat invasive cancers, whether the goal is curative or palliative. These advances are only possible because of a shared commitment toward the funding of research across the spectrum of oncology. Without it, we run the risk of stagnation, which is something no one with cancer can afford or should be asked to tolerate. As we look to the future, let us recommit to the funding of cancer research. Finally, we take pride in believing that *CA* is the *oncology journal for the nononcologist*. For, if we are to make progress in oncology together, we must speak the same language. That is the tenet that will guide *CA* as we usher in the next chapters of the journal.

## CONFLICT OF INTEREST STATEMENT

Don S. Dizon reports personal/consulting fees from Doximity and ImmunoGen Inc.; data and safety monitoring for AstraZeneca, Clovis Oncology Inc., and GlaxoSmithKline; and holds stock options in Midi outside the submitted work. Razelle Kurzrock reports support for professional activities from the Worldwide Innovative Network (WIN Consortium) for Personalized Cancer Therapy outside the submitted work. Arif H. Kamal reports personal/consulting fees from Acclivity Health and Homebase Medical outside the submitted work and is currently the chief executive officer of Prepped Health.
